# An overview of positive cultures and clinical outcomes in septic patients: a sub-analysis of the Prehospital Antibiotics Against Sepsis (PHANTASi) trial

**DOI:** 10.1186/s13054-019-2431-8

**Published:** 2019-05-21

**Authors:** Rishi S. Nannan Panday, Eline M. J. Lammers, Nadia Alam, Prabath W. B. Nanayakkara

**Affiliations:** 1 Section Acute Medicine, Department of Internal Medicine Amsterdam UMC, University Medical Centers, Location VU University Medical Center, Vrije Universiteit Amsterdam, De Boelelaan 1118, 1081HZ Amsterdam, The Netherlands; 2 Amsterdam Cardiovascular Sciences, Amsterdam UMC, University of Amsterdam, Meibergdreef 9, 1105 AZ Amsterdam, The Netherlands

**Keywords:** Sepsis, (Blood) culture, Mortality, (Multi) organ failure, Organ dysfunction, Antimicrobial therapy, Antibiotics

## Abstract

**Background:**

Sepsis remains one of the most important causes of morbidity and mortality worldwide. In approximately 30–50% of cases of suspected sepsis, no pathogen is isolated, disabling the clinician to treat the patient with targeted antimicrobial therapy. Studies investigating the differences in the patient outcomes between culture-positive and culture-negative sepsis patients have only been conducted in subgroups of sepsis patients and results are ambiguous.

**Methods:**

This is a sub-analysis of the PHANTASi (Prehospital Antibiotics Against Sepsis trial), a randomized controlled trial that focused on the effect of prehospital antibiotics in sepsis patients. We evaluated the outcome of cultures from different sources and determined what the clinical implications of having a positive culture compared to negative cultures were for patient outcomes. Furthermore, we looked at the effect of antibiotics on culture outcomes.

**Results:**

1133 patients (42.6%) with culture-positive sepsis were identified, compared to 1526 (56.4%) patients with culture-negative sepsis.

28-day mortality (RR 1.43 [95% CI 1.11–1.83]) and 90-day mortality (RR 1.41 [95% CI 1.15–1.71]) were significantly higher in culture-positive patients compared to culture-negative patients.

Culture-positive sepsis was also associated with ≥ 3 organ systems affected during the sepsis episode (RR 4.27 [95% CI 2.78–6.60]). Patients who received antibiotics at home more often had negative blood cultures (85.9% vs. 78%) than those who did not (*p* < 0.001).

**Conclusions:**

Our results show that culture-positive sepsis is associated with a higher mortality rate and culture-positive patients more often have multiple organ systems affected during the sepsis episode.

**Trial registration:**

The PHANTASi trial is registered at ClinicalTrials.gov, number NCT01988428. Date of registration: November 20, 2013.

**Electronic supplementary material:**

The online version of this article (10.1186/s13054-019-2431-8) contains supplementary material, which is available to authorized users.

## Background

Sepsis remains one of the most important causes of morbidity and mortality worldwide [1]. Every year, over 30 million persons develop sepsis of which more than five million die [2]. Although mortality rates have been decreasing, the incidence of sepsis continues to increase [2]. This is due to several factors, among which are as follows: an aging population, antibiotic resistance, improved recognition of sepsis, and use of immunotherapy [2, 3].

One of the cornerstones of the diagnosis and treatment of sepsis is the collection of (blood) cultures. This is of importance since detection of the organism that caused sepsis provides possibilities for targeted antimicrobial therapy [1]. However, culture-negative sepsis is common, and for example, Kumar et al. [4] found 29% cases of culture-negative septic shock in their cohort. Bernard et al. [5] found a negative blood culture in approximately 68% of cases with severe sepsis.

Previous studies have focused on the association between (blood) cultures and patient outcomes [6–10]. However, these studies did not produce consistent results and were only conducted in subpopulations, such as those with septic shock who were admitted to the intensive care unit (ICU) [7]. No prior studies on this subject have been conducted in septic patients who were transported by Emergency Medical Service (EMS). It is known that these septic patients who were transported by EMS are more seriously ill than those transported otherwise. Thus, sepsis patients that were transported by EMS personnel are a representative sample of the general sepsis population [11]. As these patients often require urgent care, it is quintessential to determine the relationship between (blood) culture outcomes and mortality in this group of patients in order to optimize their treatment.

Several theories have been described in literature to formulate an explanation for the low yield of micro-organisms in sepsis patients, such as prior antibiotic treatment, insufficient sampling, transport problems, and insufficient techniques. Finally, it is speculated that not all patients actually have sepsis but an alternative diagnosis [6–8]. Although previous studies have suggested that prior oral antibiotics administration might lead to decreased (blood) culture yield, limited research has been conducted to assess this association [6–8]. In an effort to concur the above sketched clinical dilemmas, the primary aims of this study were to investigate the association between culture result (positive or negative) and 28-day mortality, 90-day mortality, and the number of organ systems affected in sepsis patients. Another primary aim was to determine what the effect is of prior administration of antibiotics on culture outcome. The secondary aims of this study were to describe the association between the administration of pre-hospital antibiotics (2 g ceftriaxone in the ambulance) and mortality in culture-positive sepsis patients as well as describing the different specimen sources identified in the various types of cultures.

## Materials and methods

### Design and setting

A sub-analysis was performed using the Prehospital Antibiotics Against Sepsis (PHANTASi) trial database [12, 13]. In brief, the PHANTASi trial was a randomized controlled trial that compared the effects of training (EMS) personnel in recognizing and initiating treatment in the prehospital setting together with early administration of antibiotics for patients suspected of (severe) sepsis and septic shock when compared to usual care (fluid resuscitation and supplementary oxygen).

Patients included in the database met the following criteria: at least 18 years old, a diagnosed or suspected infection, a temperature higher than 38 °C or lower than 36 °C, and at least one other systemic inflammatory response syndrome (SIRS) criterion (heart frequency > 90/min or respiratory rate > 20 per minute, or both). An elevated or decreased leukocyte count was not included in the criteria due to the lack of pre-hospital leukocyte tests. Thus, patients were included in the study using the SEPSIS-2 criteria, as study inclusion started in 2014 before the formulation of the SEPSIS-3 criteria in 2016 [14]. Retrospective chart analysis of all charts by a panel of experts (consisting out of two acute physicians and one infectious disease specialist) was performed in order to exclude patients with an alternative diagnosis rather than sepsis [12].

### Methodology and definitions

The database consists of data from 2659 patients that were admitted to 34 different hospitals in the Netherlands from 2014 until 2016.

Culture is defined as any culture (e.g., blood, urine, sputum, wound) taken from the patient. A list of the different types of cultures analyzed can be found in Additional file 1: Table S1. Pre-hospital blood culture is referred to as a blood culture drawn in the ambulance prior to start of antibiotics in the intervention group of the PHANTASi trial. In-hospital blood culture is defined as a blood culture drawn during hospital stay. Bacteremia is defined as a positive pre-hospital and/or in-hospital blood culture. Culture-negative sepsis is defined as sepsis without any pathogen isolated from any culture. Micro-organisms in blood, cerebrospinal fluid, and sputum cultures that were very likely due to contamination were excluded from the analysis [15–17]. A list of these micro-organisms can be found in Additional file 1: Table S2. To determine the amount of organ systems involved during the sepsis episode, patients were assessed in terms of cardiovascular, respiratory, hematological, renal, hepatic, central nervous system (CNS), gastro-intestinal, and metabolic dysfunction [18]. A detailed description of the criteria can be found in Additional file 1: Table S3.

### Statistical analyses

Data-analysis was performed using R version 3.4.2. Patient characteristics are presented as frequencies and percentages for categorical variables and as means and standard deviations or median and interquartile ranges for continuous variables. To make comparisons between groups, Pearson’s chi-square tests and Wilcoxon rank-sum test were performed.

To answer the primary objective, logistic regression was performed to estimate the association between culture result (positive or negative) and respectively 28-day mortality, 90-day mortality, and the number of organ systems affected during the sepsis episode. To correct for a possible effect of confounders on the association between culture result and outcomes, additional analyses were performed using a multivariate logistic regression model that included age, group allocation, hospital location, source of infection, antibiotics at home and the total amount of blood cultures drawn. An interaction term was used to check for effect-modification of the intervention (administration of 2 g ceftriaxone in the ambulance) on the association between culture result and mortality. The number of organs affected in the sepsis episode was analyzed as a dichotomous variable (< 3 and ≥ 3 organ systems involved). To improve the robustness of our results, sensitivity analysis was performed excluding positive rectum cultures, in order to rule out the possibility of patients having a culture-positive sepsis status due to rectal colonization.

Likewise, logistic regression was performed to estimate the association between the intervention and mortality in culture-positive sepsis patients. The possible confounders ceftriaxone resistance and antibiotics at home were included in a multivariate logistic regression model. Sensitivity analysis was performed excluding urine cultures, to rule out possible asymptomatic bacteriuria.

A subgroup analysis was performed exclusively including patients that met the clinical SEPSIS-3 criteria (a quick Sequential Organ Failure Assessment (qSOFA) score of ≥ 2 in the ambulance or emergency department (ED)), assuming that baseline qSOFA score was 0 in all patients.

## Results

### Patient characteristics

A total of 2659 patients were included in the analysis. 1133 (42.6%) patients with culture-positive sepsis were identified, compared to 1526 (56.4%) patients with culture-negative sepsis. Of those, 539 (20.3%) patients had a positive blood culture and 2120 (79.7%) had a negative blood culture. Only 213 patients (13.8% of the intervention group) with a positive pre-hospital blood culture were identified. The proportion of positive pre-hospital blood cultures and the percentage of culture-positive sepsis patients differed across the different hospital locations (*p* < 0.001) (see Additional file 1: Table S17).

Patients with culture-positive sepsis had a median age of 76 years compared to 75 years for the culture-negative group (*p* = 0.029). Culture-positive sepsis patients had a higher qSOFA in both the ambulance (*p* < 0.001) and ED (*p* = 0.006). Next to that, they had a higher sepsis severity (*p* < 0.001), a longer length of stay (LOS) (*p* < 0.001), and a higher C-reactive protein (CRP) level (*p* < 0.001) than those with culture-negative sepsis (Table 1).

**Table 1:** Characteristics of culture-negative and culture-positive sepsis patients

		Culture-negative sepsis	Culture-positive sepsis	*p*
*n* (%)		1526 (57.3)	1133 (42.6)	
Group allocation(%)	Control group	612 (40.1)	520 (45.9)	0.003
	Intervention group	914 (59.9)	613 (54.1)	
Sex (%)	Female	663 (43.4)	467 (41.2)	0.267
	Male	863 (56.6)	666 (58.8)	
Age (median [IQR])		75 [65, 83]	76 [67, 83]	0.029
Charlson comorbidity index (median [IQR])		1 [1, 3]	1 [0, 3]	0.754
Antibiotics at home (%)	No	1183 (77.5)	903 (79.7)	0.193
	Yes	343 (22.5)	230 (20.3)	
qSOFA in the ambulance (%)	< 2	1189 (83.2)	803 (75.7)	< 0.001
	≥ 2	240 (16.8)	258 (24.3)	
qSOFA in the ED (%)	< 2	760 (85.5)	538 (80.1)	0.006
	≥ 2	129 (14.5)	134 (19.9)	
Sepsis severity (%)	Sepsis	648 (43.4)	353 (31.3)	< 0.001
	Severe sepsis	817 (54.8)	700 (62.1)	
	Septic shock	27 (1.8)	74 (6.6)	
Hospital LOS (median [IQR])		5 [3, 8]	7 [4, 11]	< 0.001
CRP (median [IQR])		68 [29, 149]	93 [34, 189]	< 0.001
Source of infection (%)	CNS	7 (0.5)	4 (0.4)	< 0.001
	Intra-abdominal	82 (5.9)	95 (8.9)	
	Line	1 (0.1)	3 (0.3)	
	Pulmonal	1019 (73.4)	448 (41.9)	
	Skin/tissue	66 (4.8)	78 (7.3)	
	Urinary tract	175 (12.6)	398 (37.3)	
	Other	38 (2.7)	42 (3.9)	
Total number of blood cultures (median [IQR])		2 [1, 2]	2 [2, 4]	< 0.001

*qSOFA* quick Sequential Organ Failure Assessment score, *ED* emergency department, *LOS* length of stay, *CRP* C-reactive protein, *CNS* central nervous system

Suspected sources of infection differed between the culture-negative and culture-positive groups (*p* < 0.001). For example, urinary tract infection was more common in the culture-positive group (37.3% vs. 12.6%), whereas respiratory tract infection was more often seen in culture-negative sepsis patients (73.4% vs. 41.9%). The median amount of blood cultures drawn was 2 in both groups, although interquartile ranges differed (*p* < 0.001) (see Table 1 for further details). Patient characteristics for culture-negative and culture-positive patients stratified by group allocation can be found in (Additional file 1: Table S4).

In the subgroup of patients with culture-positive sepsis, patients in the intervention group had a higher qSOFA score in the ambulance (*p* = 0.004) and also a higher sepsis severity (*p* = 0.051) compared to the control group (Additional file 1: Table S5).

### Mortality

**Fig. 1:**
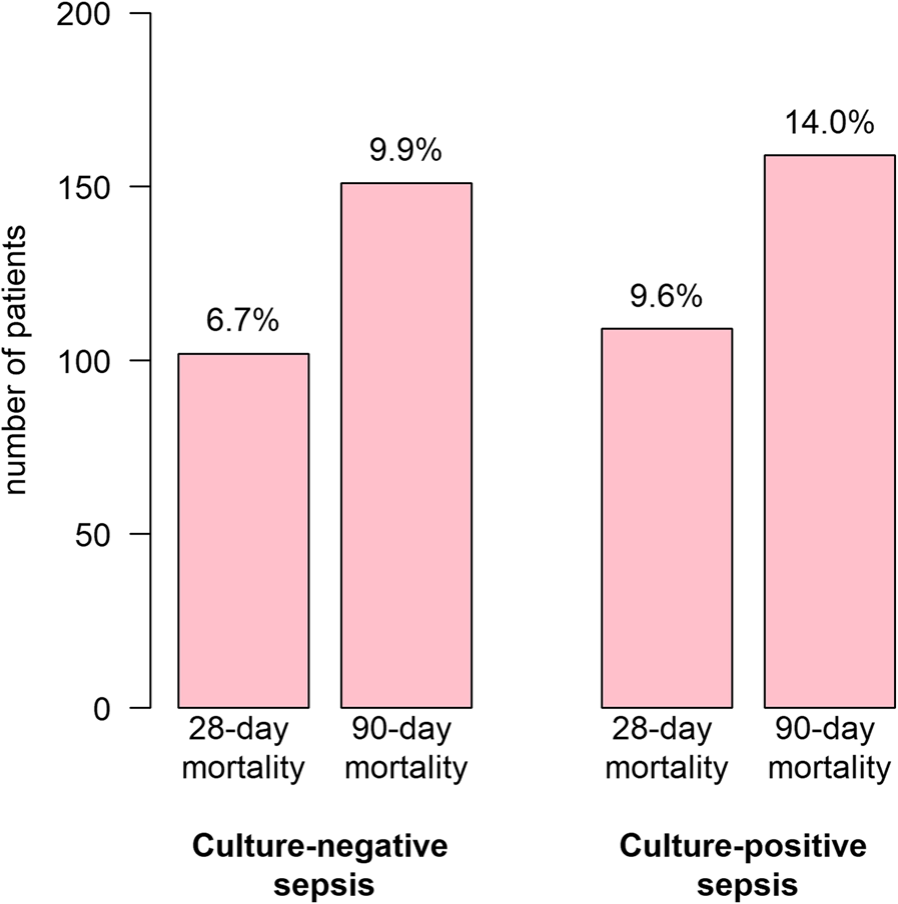
Mortality in culture-positive and culture-negative sepsis

Culture-positive patients had a higher 28-day mortality (9.6% vs. 6.7%) and 90-day mortality (14% vs. 9.9%) than culture-negative patients (Fig. 1). Patients with a positive blood culture had a higher 28-day mortality (10.4% vs. 7.3%) and 90-day mortality (15.1% vs. 10.8%) than those who did not have a positive blood culture (Table 2).

**Table 2:** Mortality for patients with a positive (blood) culture

	Culture-negative sepsis			Culture-positive sepsis			*p* (total CNS vs CPS)
	Control group	Intervention group	Total	Control group	Intervention group	Total	
*n*	612	914	1526	520	613	1133	
28-day mortality	46 (7.5)	56 (6.1)	102 (6.7)	46 (8.9)	63 (10.3)	109 (9.6)	0.007
90-day mortality	63 (10.3)	88 (9.6)	151 (9.9)	70 (13.5)	89 (14.5)	159 (14)	0.001
	Non-bacteremic			Bacteremic			*p* (total NB vs B)
	Control group	Intervention group	Total	Control group	Intervention group	Total	
*n*	909	1211	2120	223	316	539	
28-day mortality	74 (8.1)	81 (6.7)	155 (7.3)	18 (8.1)	38 (12)	56 (10.4)	0.022
90-day mortality	104 (11.4)	125 (10.3)	229 (10.8)	29 (13.1)	52 (16.5)	81 (15.1)	0.008

*CNS* culture-negative sepsis, *CPS* culture-positive sepsis, *NB* non-bacteremic, *B* bacteremic

Culture-positive sepsis was associated with a higher 28-day (RR 1.43 [95% CI 1.11–1.83]) and 90-day mortality (RR 1.41 [95% CI 1.15–1.71]). Bacteremia (positive pre-hospital and/or in-hospital blood culture) was associated with a higher 28-day mortality (RR 1.42 [95% CI 1.06–1.87]) and a higher 90-day mortality (RR 1.39 [95% CI 1.09–1.73]). Similar results were found in the group of patients with a positive pre-hospital blood culture, with an RR of 1.82 [95% CI 1.24–2.55] for 28-day mortality and with an RR of 1.60 [95% CI 1.16–2.12] for 90-day mortality. Results were consistent after correction for possible confounders in a multivariate regression model (see Table 3). Sensitivity analyses after exclusion of positive rectum cultures showed similar relative risks (see Additional file 1: Table S13). A significant association between a positive in-hospital blood culture and mortality was exclusively found in the multivariate analysis with an RR of 1.56 [95% CI 1.05–2.25] for 28-day mortality and an RR of 1.45 [95% CI 1.05–1.95] for 90-day mortality.

**Table 3:** The association between a positive (blood) culture and mortality

	*p*	RR	95% CI
Positive pre-hospital blood culture			
	Unadjusted analysis		
28-day mortality	0.002	1.82	1.24–2.55
90-day mortality	0.004	1.60	1.16–2.12
	Adjusted analysis^∞^		
28-day mortality	< 0.001	2.10	1.36–3.09
90-day mortality	0.004	1.71	1.19–2.37
Positive in-hospital blood culture			
	Unadjusted analysis		
28-day mortality	0.115	1.31	0.92–1.68
90-day mortality	0.059	1.30	0.98–1.68
	Adjusted analysis^∞^		
28-day mortality	0.025	1.56	1.05–2.25
90-day mortality	0.022	1.45	1.05–1.95
Bacteremia (positive pre-hospital and/or in-hospital blood culture)			
	Unadjusted analysis		
28-day mortality	0.018	1.42	1.06–1.87
90-day mortality	0.006	1.39	1.09–1.73
	Adjusted analysis^∞^		
28-day mortality	0.002	1.71	1.22–2.34
90-day mortality	0.002	1.55	1.18–1.99
Culture-positive sepsis (with or without bacteremia)			
	Unadjusted analysis		
28-day mortality	0.006	1.43	1.11–1.83
90-day mortality	0.001	1.41	1.15–1.71
	Adjusted analysis^∞^		
28-day mortality	0.004	1.54	1.15–2.03
90-day mortality	< 0.001	1.53	1.21–1.91

^∞^Adjusted for age, group allocation, antibiotics at home, hospital location, source of infection, and total amount of blood cultures drawn

Subgroup analysis of patients fulfilling clinical SEPSIS-3 criteria showed a significant association between bacteremia and 90-day mortality, with a RR of 1.54 [95% CI 1.01–1.83] in the unadjusted analysis and a RR of 1.51 [95% CI 1.03–2.08] in the adjusted analysis. No significant association was found for between positive blood culture and 28-day mortality; neither were significant associations found for patients with culture-positive sepsis. Further details can be found in Additional file 1: Table S21. 55.2% of patients not fulfilling clinical SEPSIS-3 criteria, that is a qSOFA score < 2, had dysfunction of at least 1 organ system (see Additional file 1: Table S22).

### Organ failure

The median number of organ systems affected was one in both the culture-negative and culture-positive groups (*p* < 0.001). Culture-positive patients were more likely to have cardiovascular (*p* < 0.001), renal (*p* < 0.001), hepatic (*p* < 0.001), CNS (*p* < 0.001), and metabolic (*p* < 0.001) dysfunction.

**Fig. 2:**
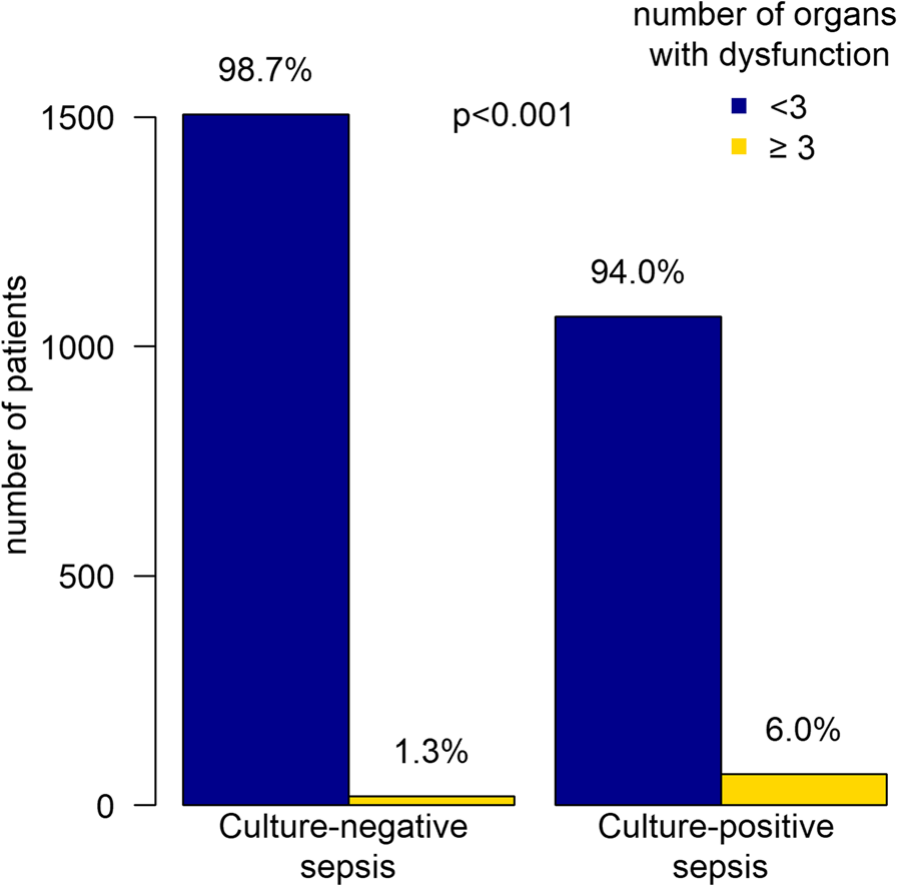
Organ dysfunction in culture-positive and culture-negative sepsis

Both culture-positive sepsis and bacteremia were positively associated with ≥ 3 organ systems affected during the sepsis episode (RR 4.27 [95% CI 2.78–6.60] and RR 2.68 [95% CI 1.78–3.94]) (Fig. 2). After correction for possible confounders, an RR of 4.05 [95% CI 2.47–6.63] for culture-positive sepsis and an RR of 2.10 [95% CI 1.28–3.36] for bacteremia were found.

### Antibiotics at home

85.9% of patients in the group with antibiotics at home had a negative blood culture versus 78% in the group without (*p* < 0.001). No significant association was found between prior administration of antibiotics at home with pre-hospital blood-culture result and the result of any culture (see Additional file 1: Table S9).

### Pre-hospital antibiotics in culture-positive sepsis

No association was observed between the administration of pre-hospital antibiotics and 28-day (RR 1.16 [95% CI 0.81–1.64]) and 90-day mortality (RR 1.08 [95% CI: 0.80–1.42]) in the subgroup of patients with culture-positive sepsis. Neither was this association seen in the corrected analysis (see Additional file 1: Table S10). Sensitivity analysis after exclusion of positive urine cultures showed similar relative risks (see Additional file 1: Table S14).

### Specimen sources and reported ceftriaxone resistance

The three most common bacteria isolated from blood cultures were *Escherichia coli*, *Streptococcus pneumoniae*, and *Staphylococcus aureus*. An overview of the 10 most common pathogens identified in blood, sputum, urine, and wound/skin/soft tissue cultures and related mortality rates can be found in the Appendix (Additional file 1: Table S15–S20). In our cohort, a rate of 12.9% of ceftriaxone resistance was seen in culture-positive patients. Higher mortality rates were seen in patients that were ceftriaxone-resistant both in the intervention and control group (see Additional file 1: Table S11). The pathogens that were most commonly found resistant to ceftriaxone were *Enterococcus faecalis*, *Pseudomonas aeruginosa*, and *E. coli*.

## Discussion

Patients, who had a culture-positive sepsis, had a significantly higher mortality rate than those who had a culture-negative sepsis. We found culture-negative sepsis in 56.4%, and this incidence in our cohort was higher than that seen in earlier studies that describe incidences of 30.6–47.1% [6–8]. Several factors might have led to this difference in the outcome. We excluded cultures that were most likely positive due to contamination. Apart from this, we included all sepsis patients, and not only those with severe sepsis or sepsis shock. This in turn might have led to our patients having a lower bacterial burden and thereby making it more difficult to detect a pathogen with a conventional culture. Another possible explanation is that the administration of pre-hospital antibiotics in the intervention group contributed to the higher rate of culture-negative sepsis. Culture-negative sepsis was seen more often in the intervention group compared to the control group (59.2% vs 53.9%).

When comparing culture-positive patients with culture-negative ones, we saw several notable differences between these groups. Culture-positive patients had higher qSOFA scores, a higher severity of illness, a longer hospital LOS, and a higher level of the CRP. These clinical parameters suggest that culture-positive patients might have been sicker than culture-negative patients. Furthermore, higher mortality rates were found in culture-positive patients compared to culture-negative patients, and the culture-positive patients more often had dysfunction of ≥ 3 organ systems. All these findings suggest a higher burden of disease in the culture-positive group. Although we cannot completely rule out the possibility of patients having a positive culture status due to colonization of specimens in non-sterile culture sites, our findings were confirmed by a sensitivity analysis after exclusion of positive rectum cultures.

Several earlier studies were conducted on the association between culture-positive status in sepsis patients and mortality. Like in our cohort, Kethireddy et al. [7] found less impressive clinical parameters in culture-negative septic shock patients compared to culture-positive counterparts. However, survival was similar in culture-negative patients and culture-positive patients. Interestingly, a delayed administration of antibiotics led to a similar increase in mortality in both groups, supporting the hypothesis that culture-negative sepsis patients do not have an alternative non-infectious diagnosis. Gupta et al. [6] also looked at the association between culture status and mortality and found a higher mortality rate in culture-negative severe sepsis patients. A possible explanation given is the lack of guidance for specific antibiotic treatment in culture-negative patients. However, as selection of culture-positive patients was based on codes in patient records, undercoding of specific pathogens might have led to incorrect classification of patients into the culture-negative group [6]. Like in our study, Phua et al. [8] found higher severity of illness, a longer LOS, and more organ dysfunction in culture-positive severe sepsis compared to culture-negative severe sepsis. However, no independent association between a positive culture and mortality was found in a multivariate model. As the authors describe this model included all covariates available at baseline, inclusion of covariates that are to be part of the causal pathway of sepsis might have led to overadjustment [8, 19]. Therefore, we chose not to include sepsis severity in the multivariate model, although previous work has shown that the probability of finding bacteremia is dependent on the clinical context [20].

Although we think it is unlikely that patients in our cohort suffered from alternative non-infectious diagnoses because of strict inclusion criteria and retrospective chart analysis by an expert panel, study inclusion was carried out before formulation of the SEPSIS-3 criteria. Therefore, we included patients that do not fulfill clinical SEPSIS-3 criteria and do not meet the current definition of sepsis.

A possible explanation for the low mortality rates seen our cohort, next to the fact that other studies have been conducted in cohorts of patients with a higher severity of disease only (severe sepsis or septic shock), is that all EMS personnel participating in the PHANTASi trial was trained to recognize sepsis in a timely manner. Apart from this, awareness of sepsis was improved during the study period through the use of several media. This led to better recognition and improved treatment.

In the subgroup of patients that met SEPSIS-3 criteria, we only found a significant association between a positive blood culture and 90-day mortality. We can however not see these specific findings in the light of other literature, as no research has been performed in the field of culture positivity in the whole population of sepsis patients after formulation of SEPSIS-3 criteria. In our cohort, we found that 55.2% of patients that did not meet clinical SEPSIS-3 criteria (qSOFA < 2) had organ dysfunction of one or more organ systems (Table 4). Meta-analysis has shown a low sensitivity of the qSOFA score in screening for mortality outside of the ICU [21]. In a recent article, the same authors pray for utilization of SIRS criteria outside of the ICU [22]. Therefore, we do not exclude the possibility of review of the use of qSOFA score as a clinical criterion for sepsis, as fast recognition of sepsis is found to reduce mortality [23].

**Table 4:** Organ dysfunction in culture-negative and culture-positive patients

	Culture-negative sepsis			Culture-positive sepsis			
	Control group	Intervention group	Total	Control group	Intervention group	Total	*p* (Total CNS vs. CPS)
*n*	612	914	1526	520	613	1133	
Number of organ systems affected (median [IQR])	1 [0, 1]	1 [0, 1]	1 [0, 1]	1 [0, 2]	1 [0, 2]	1 [0, 2]	< 0.001
Cardiovascular dysfunction (*n* (%))	51 (8.5)	84 (9.3)	135 (9)	78 (15.2)	128 (21.2)	206 (18.9)	< 0.001
Respiratory dysfunction (*n* (%))	215 (35.7)	307 (33.9)	522 (34.6)	163 (31.6)	230 (38)	393 (35)	0.849
Hematological dysfunction (*n* (%))	9 (1.5)	12 (1.3)	21 (1.4)	6 (1.2)	13 (2.2)	19 (1.7)	0.644
Renal dysfunction (*n* (%))	29 (4.8)	41 (4.5)	70 (4.7)	50 (9.7)	77 (12.7)	127 (11.3)	< 0.001
Hepatic dysfunction (*n* (%))	13 (2.2)	11 (1.2)	24 (1.6)	28 (5.4)	29 (4.8)	57 (5.1)	< 0.001
CNS dysfunction (*n* (%))	110 (18.3)	177 (19.6)	287 (19.1)	128 (24.8)	161 (26.7)	289 (25.8)	< 0.001
Gastro-intestinal dysfunction (*n* (%))	2 (0.3)	5 (0.6)	7 (0.5)	2 (0.4)	3 (0.5)	5 (0.4)	1.000
Metabolic dysfunction (*n* (%))	98 (16.3)	137 (15.1)	235 (15.6)	151 (29.2)	166 (27.3)	317 (28.2)	< 0.001

*CNS* central nervous system or culture-negative sepsis, *CPS* culture-positive sepsis

Insufficient techniques may have contributed to the high amount culture-negative cases of sepsis seen in general. Bloos et al. found a positive PCR (polymerase chain reaction) in approximately 20% of culture-negative sepsis patients [24]. Earlier studies have showed that multiplex real-time PCR leads to a faster diagnosis and to a reduction of the amount of days of inadequate antibiotic treatment [25–27].

Quick adequate antimicrobial therapy is of great importance as long-term treatment with broad-spectrum antibiotics leads to resistant micro-organisms and is associated with superinfections with *C. difficile* and fungi [28].

We found that culture-negative sepsis was most common in respiratory tract infection and that urinary tract infection was most common in culture-positive sepsis. These findings were similar to those by Phua et al. [8]. A possible explanation could be that sepsis due to pneumonia is more often caused by viruses and/or fungi, which is not routinely tested for in most hospitals. There was a variability in culture positivity across the different hospital sites. This is probably due to the differences in pre-analytical (the time from collection to incubation) between hospital locations. Some hospitals are located in rural areas and others in urban areas, where driving time to the closest hospital is usually shorter. Earlier research has shown that longer pre-analytical time leads to lower blood culture positivity [29]. Moreover, guidelines for antibiotic treatment differ between different hospital location, which might also influence culture results.

Of note, in culture-positive sepsis patients, higher mortality rates were observed in the intervention group compared to the control group. A possible explanation for this could be that EMS personnel deliberately assigned the intervention group protocol to sicker patients.

An association between prior administration of antibiotics at home and a negative pre-hospital culture was not seen. However, as the amount of patients with a positive pre-hospital blood culture was low (13.8% of patients in the invention group), we cannot exclude the possibility that due to obtainment of a culture early in the disease process, a lower disease burden at that time may have influenced the results.

Our study contains several strengths. Firstly, to the best of our knowledge, this is the first study which investigates culture outcomes in all sepsis patients, instead of a subset of patients [6–8]. In addition, we excluded cultures that were likely false-positive due to contamination which was not done in two of the previously mentioned studies [6, 7].

Furthermore, we used data of a large multi-center trial that consisted of patients from both urban and rural areas which allow our results to be extrapolated easier to other countries.

Our study also holds limitations. First, we did not have data on the levels of the pro-calcitonin. This would have yielded extra information, as this biomarkers is found superior to the CRP for the diagnosis of sepsis in most studies [30].

Secondly, choosing a cut-off of 3 in analyzing the amount of organ dysfunction might be considered somewhat arbitrary. However, earlier research has shown that involvement of ≥ 3 organ systems is associated with an in-hospital mortality rate of more than 50% [31].

## Conclusions

Our results show that culture-positive sepsis is associated with a higher mortality rate and that culture-positive sepsis patients more often have ≥ 3 organ systems affected during the sepsis episode. Future studies should focus on the etiological mechanisms that lead to the differences in culture outcomes in sepsis patients in order to further reduce mortality rates of sepsis.

## Additional file

Additional file 1: Supplementary information on patient characteristics and culture outcomes. (DOCX 52 kb)

## Abbreviations

CNS: Central nervous system or culture-negative sepsis
CPS: Culture-positive sepsis
CRP: C-reactive protein
ED: Emergency department
EMS: Emergency medical service
ICU: Intensive care unit
LOS: Length of stay
PCR: Polymerase chain reaction
PHANTASi trial: Prehospital Antibiotics Against Sepsis trial
qSOFA: Quick Sequential Organ Failure Assessment score
SIRS criteria: Systemic inflammatory response syndrome criteria: temperature > 38 °C or < 36 °C; heart rate > 90 beats per minute; respiratory rate > 20 breaths per minute or PaCO_2_ < 32 mmHg; and white blood cell count > 12,000/cu mm, < 4000/cu mm, or > 10% immature (band) forms (according to sepsis 3 criteria)
