# Supplementary material for: An overview of positive cultures and clinical outcomes in septic patients: a sub-analysis of the Prehospital Antibiotics Against Sepsis (PHANTASi) trial
Source: Crit Care. 2019 May 21;23:182. doi: 10.1186/s13054-019-2431-8 (PMC6530106; doi:10.1186/s13054-019-2431-8)
Supplement: Supplementary file 1 — Supplementary information on patient characteristics and culture outcomes. (DOCX 52 kb) [file 13054_2019_2431_MOESM1_ESM.docx]

**Additional file 1**

| **Table S1: Types of cultures/ other microbiological diagnostics obtained from the included patients that were included in the analysis** |
| --- |
| Pre-hospital blood culture (in the ambulance)  In-hospital blood culture  Urine culture  Sputum culture  Wound/ skin/ soft tissue culture  Pleural fluid culture  Ascites culture  Culture of fluid obtained by bronchoalveolar lavage (BAL)  Culture of synovial fluid obtained by joint puncture  Culture of intra-corporal material  Feces culture  Cerebrospinal fluid culture  Pharynx culture (incl. swab for Influenza)  Rectum culture  Perineum culture |

| **Table S2: List of pathogens that are likely due to contamination(1-3)** | |
| --- | --- |
| Blood and central nervous system cultures | Sputum cultures |
| Micrococcus species  Bacillus species other than B. anthracis  Coagulase-negative staphylococci  Corynebacterium species  Propionibacterium acnes | Candida albicans |

| **Table S3: List of the used criteria for organ dysfunction (4)** | |
| --- | --- |
| Cardiovascular dysfunction | Decrease of SBP > 40 mmHg and/or SBP <90 or MAP ≤ 65 (despite adequate fluid resuscitation) |
| Respiratory dysfunction | Arterial hypoxemia (arterial oxygen tension [PaO2]/fraction of inspired oxygen [FiO2] <300) |
| Hematological dysfunction | Coagulation abnormalities (international normalized ratio [INR] >1.5 and/or activated partialthromboplastin time [aPTT] >60 seconds) and/or Thrombocytopenia (platelet count <100,000 microL/L) |
| Renal dysfunction | Acute oliguria (urine output <0.5 mL/kg/hr for at least two hours despite adequate fluid resuscitation) and/or Creatinine increase >0.5 mg/dL or 44.2 micromol/L |
| Hepatic dysfunction | Hyperbilirubinemia (plasma total bilirubin >4 mg/dL or 70 micromol/L) |
| CNS (central nervous system) dysfuntion | Altered mental status |
| Gastro-intestinal dysfunction | Ileus (absent bowel sounds) |
| Metabolic dysfunction | Hyperlactatemia (>2 mmol/L) |

| **Table S4: Characteristics of culture-negative and culture-positive sepsis patients stratified by group allocation** | | | | | | |
| --- | --- | --- | --- | --- | --- | --- |
|  |  | Culture-negative sepsis | | Culture-positive sepsis | |  |
|  |  | Control group | Intervention group | Control group | Intervention group | p |
| n |  | 612 | 914 | 520 | 613 |  |
| sex (%) | female | 263 (43) | 400 (43.8) | 222 (42.7) | 245 (40) | 0.518 |
|  | male | 349 (57) | 514 (56.2) | 298 (57.3) | 368 (60) |  |
| age (median [IQR]) |  | 75 [64, 82] | 75.5 [65, 83] | 76 [66, 83] | 77 [67, 83] | 0.128 |
| Charlson comorbidity index (median [IQR]) |  | 1 [1, 2] | 1 [1, 3] | 1 [1, 3] | 1 [0, 3] | 0.253 |
| Antibiotics at home (%) | no | 463 (75.7) | 720 (78.8) | 416 (80) | 487 (79.4) | 0.263 |
|  | yes | 149 (24.3) | 194 (21.2) | 104 (20) | 126 (20.6) |  |
| qSOFA in the ambulance (%) | <2 | 486 (85.3) | 703 (81.8) | 382 (79.9) | 421 (72.2) | <0.001 |
|  | ≥ 2 | 84 (14.7) | 156 (18.2) | 96 (20.1) | 162 (27.8) |  |
| qSOFA in the ED (%) | <2 | 295 (81.9) | 465 (87.9) | 245 (81.4) | 293 (79) | 0.003 |
|  | ≥ 2 | 65 (18.1) | 64 (12.1) | 56 (18.6) | 78 (21) |  |
| sepsis severity (%) | sepsis | 250 (42.1) | 398 (44.3) | 174 (33.5) | 179 (29.4) | <0.001 |
|  | severe sepsis | 333 (56.1) | 484 (53.9) | 320 (61.7) | 380 (62.5) |  |
|  | septic shock | 11 ( 1.9) | 16 ( 1.8) | 25 ( 4.8) | 49 ( 8.1) |  |
| hospital LOS (median [IQR]) |  | 5 [3, 8] | 5 [3, 8] | 6 [4, 11] | 7 [4, 12] | <0.001 |
| CRP (median [IQR]) |  | 70 [25, 160.25] | 67 [32, 141] | 89 [32, 190] | 95 [37, 189] | <0.001 |
| source of infection (%) | CNS* | 1 ( 0.2) | 6 ( 0.7) | 1 ( 0.2) | 3 ( 0.5) | <0.001 |
|  | intra-abdominal | 29 ( 5.3) | 53 ( 6.3) | 35 ( 7.1) | 60 (10.5) |  |
|  | line | 0 ( 0) | 1 ( 0.1) | 2 ( 0.4) | 1 ( 0.2) |  |
|  | pulmonal | 415 (76) | 604 (71.7) | 214 (43.1) | 234 (40.9) |  |
|  | skin/tissue | 22 ( 4) | 44 ( 5.2) | 34 ( 6.9) | 44 ( 7.7) |  |
|  | urinary tract | 65 (11.9) | 110 (13.1) | 191 (38.5) | 207 (36.2) |  |
|  | other | 14 ( 2.6) | 24 ( 2.9) | 19 ( 3.8) | 23 ( 4) |  |
| total number of blood cultures (median [IQR]) |  | 2 [2, 2] | 2 [1, 2] | 2 [2, 3] | 2 [2, 4] | <0.001 |
| *Used abbreviations: qSOFA = quick Sequential Organ Failure Assessment score, ED = emergency department, LOS = length of stay, CRP = C-reactive protein, CNS = central nervous system | | | | | | |

| **Table S5: Characteristics of the control group and the intervention group in the subgroup of patients with culture-positive sepsis** | | | | |
| --- | --- | --- | --- | --- |
|  |  | control group | intervention group | p |
| n |  | 520 | 613 |  |
| sex (%) | female | 222 (42.7) | 245 (40) | 0.385 |
|  | male | 298 (57.3) | 368 (60) |  |
| age (median [IQR]) |  | 76 [66, 83] | 77 [67, 83] | 0.508 |
| Charlson Comorbidity Index (median [IQR]) | | 1 [1, 3] | 1 [0, 3] | 0.105 |
| Antibiotics at home (%) | no | 416 (80) | 487 (79.4) | 0.875 |
|  | yes | 104 (20) | 126 (20.6) |  |
| qSOFA in the ambulance(%) | <2 | 382 (79.9) | 421 (72.2) | 0.005 |
|  | ≥ 2 | 96 (20.1) | 162 (27.8) |  |
| qSOFA in the ED (%) | <2 | 245 (81.4) | 293 (79) | 0.494 |
|  | ≥ 2 | 56 (18.6) | 78 (21) |  |
| Ceftriaxone resistance (%) | no | 455 (87.5) | 532 (86.8) | 0.788 |
|  | yes | 65 (12.5) | 81 (13.2) |  |
| Sepsis severity (%) | sepsis | 174 (33.5) | 179 (29.4) | 0.050 |
|  | severe sepsis | 320 (61.7) | 380 (62.5) |  |
|  | septic shock | 25 ( 4.8) | 49 ( 8.1) |  |
| hospital LOS (median [IQR]) |  | 6 [4, 11] | 7 [4, 12] | 0.060 |
| CRP (median [IQR]) |  | 89 [32, 190] | 95 [37, 189] | 0.435 |
| Source of infection (%) | CNS* | 1 ( 0.2) | 3 ( 0.5) | 0.454 |
|  | intra-abdominal | 35 ( 7.1) | 60 (10.5) |  |
|  | line | 2 ( 0.4) | 1 ( 0.2) |  |
|  | pulmonal | 214 (43.1) | 234 (40.9) |  |
|  | skin/tissue | 34 ( 6.9) | 44 ( 7.7) |  |
|  | urinary tract | 191 (38.5) | 207 (36.2) |  |
|  | other | 19 ( 3.8) | 23 ( 4) |  |
| Total amount of blood cultures drawn (median [IQR]) | | 2 [2, 3] | 2 [2, 4] | 0.659 |
| *Used abbreviations: CNS = central nervous system | | | | |

| **Table S6: Characteristics of patients without prior administration of antibiotics at home and those with prior administration of antibiotics at home** | | | | |
| --- | --- | --- | --- | --- |
|  |  | No prior administration antibiotics at home | Prior administration of antibiotics at home | p |
| n |  | 2086 | 573 |  |
| Sex (%) | female | 872 (41.8) | 258 (45) | 0.182 |
|  | male | 1214 (58.2) | 315 (55) |  |
| Age (median [IQR]) |  | 75 [65, 83] | 76 [67, 83] | 0.409 |
| Charlson Comorbity Index (median [IQR]) |  | 1 [1, 3] | 2 [1, 3] | 0.080 |
| qSOFA* in the ambulance (%) | ≥2 | 380 (19.4) | 118 (22.1) | 0.192 |
|  | <2 | 1576 (80.6) | 416 (77.9) |  |
| qSOFA* in the ED* (%) | ≥2 | 201 (16.2) | 62 (19.2) | 0.237 |
|  | <2 | 1037 (83.8) | 261 (80.8) |  |
| Ceftriaxone resistance(%) | no | 1971 (94.5) | 538 (93.9) | 0.656 |
|  | yes | 115 ( 5.5) | 35 ( 6.1) |  |
| Sepsis severity (%) | sepsis | 796 (38.8) | 205 (36.3) | 0.302 |
|  | severe sepsis | 1175 (57.2) | 342 (60.5) |  |
|  | septic shock | 83 ( 4) | 18 ( 3.2) |  |
| Hospital LOS* (median [IQR]) |  | 6 [4, 9] | 6 [4, 10] | 0.002 |
| CRP* (median [IQR]) |  | 77 [30.75, 166.5] | 78 [35.5, 172] | 0.352 |
| Number of organ systems affected (median[IQR]) |  | 1[0, 2] | 1 [0, 2] | 0.783 |
| *Used abbreviations: qSOFA = quick Sequential Organ Failure Assessment score, ED = emergency department, LOS = length of stay, CRP = C-reactive protein | | | | |

| **Table S7: Organ dysfunction in non-bacteremic and bacteremic patients** | | | | | | | |
| --- | --- | --- | --- | --- | --- | --- | --- |
|  | Non-bacteremic | | | Bacteremic | | |  |
|  | Control  group | Intervention group | Total | Control group | Intervention  group | Total | p (Total NB* vs. B*) |
| n | 909 | 1211 | 2120 | 223 | 316 | 539 |  |
| Number of organ systems affected (median [IQR]) | 1 [0, 1] | 1 [0, 1] | 1 [0, 1] | 1 [0, 2] | 1 [0, 2] | 1 [0, 2] | <0.001 |
| Cardiovascular dysfunction (n (%)) | 84  ( 9.4) | 139 (11.6) | 223 (10.6) | 45 (20.5) | 73 (23.5) | 118 (22.2) | <0.001 |
| Respiratory dysfunction (n (%)) | 321 (35.7) | 442 (36.8) | 763 (36.4) | 57 (25.8) | 95 (30.4) | 152 (28.5) | 0.001 |
| Hematological dysfunction (n (%)) | 11  ( 1.2) | 18 ( 1.5) | 29  ( 1.4) | 4 ( 1.8) | 7 ( 2.3) | 11  ( 2.1) | 0.344 |
| Renal dysfunction (n (%)) | 52  ( 5.8) | 73 ( 6.1) | 125  ( 6) | 27 (12.2) | 45 (14.4) | 72 (13.5) | <0.001 |
| Hepatic dysfunction (n (%)) | 24  ( 2.7) | 15 ( 1.3) | 39  ( 1.9) | 17  ( 7.7) | 25 ( 8) | 42  ( 7.9) | <0.001 |
| CNS* dysfunction (n (%)) | 177 (19.7) | 258 (21.6) | 435 (20.8) | 61 (27.6) | 80 (25.7) | 141 (26.5) | 0.005 |
| Gastro-intestinal dysfunction (n (%)) | 4 ( 0.4) | 6 ( 0.5) | 10  ( 0.5) | 0 ( 0) | 2 ( 0.6) | 2  ( 0.4) | 1.000 |
| Metabolic dysfunction (n (%)) | 162 (18) | 194 (16.2) | 356 (17) | 87 (39.2) | 109 (34.7) | 196 (36.6) | <0.001 |
| *Used abbreviation: CNS = central nervous system, NB = non-bacteremic, B = bacteremic | | | | | | | |

| **Table S8: The association between a positive (blood) culture and organ dysfunction (≥3 organ systems affected)** | | | | |
| --- | --- | --- | --- | --- |
|  | p | RR | | 95% CI |
|  | Positive pre-hospital blood culture | | | |
| Unadjusted analysis | 0.002 | 2.35 | 1.32-3.84 | |
| Adjusted analysis ∞ | 0.063 | 1.86 | 0.94-3.42 | |
|  | Positive in-hospital blood culture | | | |
| Unadjusted analysis | <0.001 | 2.84 | 1.83-4.24 | |
| Adjusted analysis ∞ | 0.001 | 2.47 | 1.45-4.04 | |
|  | Bacteremia (positive pre-hospital and/or in-hospital blood culture) | | | |
| Unadjusted analysis | <0.001 | 2.68 | | 1.78-3.94 |
| Adjusted analysis ∞ | 0.003 | 2.10 | | 1.28-3.36 |
|  | Culture-positive sepsis (with or without bacteraemia) | | | |
| Unadjusted analysis | <0.001 | 4.27 | | 2.78-6.60 |
| Adjusted analysis ∞ | <0.001 | 4.05 | | 2.47-6.63 |
| ∞ Adjusted for age, group allocation, antibiotics at home, hospital location, source of infection and total amount of blood cultures drawn | | | | |

| **Table S9: Culture-positivity for patients without and with prior administration of antibiotics at home** | | | |  |
| --- | --- | --- | --- | --- |
|  |  | No antibiotics at home | Antibiotics at home | p |
| n |  | 2086 | 573 |  |
| Pre-hospital blood culture result (%) | negative | 1910 (91.6) | 536 (93.5) | 0.144 |
|  | positive | 176 ( 8.4) | 37 ( 6.5) |  |
| In-hospital blood culture result (%) | negative | 1770 (84.9) | 518 (90.4) | 0.001 |
|  | positive | 316 (15.1) | 55 ( 9.6) |  |
| Blood culture result (pre-hospital and/or in-hospital) (%) | negative | 1628 (78) | 492 (85.9) | <0.001 |
|  | positive | 458 (22) | 81 (14.1) |  |
| Any culture result (%) | negative | 1183 (56.7) | 343 (59.9) | 0.193 |
|  | positive | 903 (43.3) | 230 (40.1) |  |

| **Table S10: The association between the administration of pre-hospital antibiotics in the ambulance and mortality in the subgroup of patients with culture-positive sepsis** | | | |
| --- | --- | --- | --- |
|  | p | RR | 95% CI |
|  | 28-day mortality | | |
| Unadjusted analysis | 0.422 | 1.16 | 0.81-1.64 |
| Adjusted analysis ∞ | 0.435 | 1.15 | 0.80-1.64 |
|  | 90-day mortality | | |
| Unadjusted analysis | 0.619 | 1.08 | 0.80-1.42 |
| Adjusted analysis ∞ | 0.647 | 1.07 | 0.80-1.42 |
| ∞ Adjusted for ceftriaxone resistance and antibiotics at home | | | |

| **Table S11: 28-day and 90-day mortality for the control group and the intervention, stratified by ceftriaxone resistance** | | | | | |
| --- | --- | --- | --- | --- | --- |
|  | Control group | | Intervention group | | p (comparison of all 4 groups) |
|  | Ceftriaxone resistant | | Ceftriaxone resistant | |  |
|  | No | Yes | No | Yes |  |
| n | 1067 | 65 | 1442 | 85 |  |
| 28-day mortality (n, %) | 83 ( 7.8) | 9 (13.8) | 107 ( 7.4) | 12 (14.1) | 0.044 |
|  | p = 0.133 | | p = 0.042 | |  |
| 90-day mortality (n, %) | 119 (11.2) | 14 (21.5) | 155 (10.7) | 22 (25.9) | <0.001 |
|  | p = 0.020 | | p < 0.001 | |  |

| **Table S12: Culture positivity stratified by hospital location** | | | | | |
| --- | --- | --- | --- | --- | --- |
| Hospital name | n | Culture-positive sepsis (%) | Positive blood culture (%) | Positive in-hospital blood culture (%) | Positive pre-hospital blood culture (%) |
| Albert Schweitzer | 586 | 261 (44.5) | 126 (21.5) | 86 (14.7) | 54 ( 9.2) |
| Amsterdam UMC, location AMC | 84 | 42 (50) | 18 (21.4) | 10 (11.9) | 10 (11.9) |
| Amphia | 111 | 54 (48.6) | 21 (18.9) | 12 (10.8) | 12 (10.8) |
| Amstelland | 42 | 20 (47.6) | 9 (21.4) | 6 (14.3) | 3 ( 7.1) |
| Beatrix | 51 | 25 (49) | 13 (25.5) | 7 (13.7) | 7 (13.7) |
| Bovenij | 77 | 28 (36.4) | 18 (23.4) | 15 (19.5) | 4 ( 5.2) |
| Bravis, location Bergen op Zoom | 21 | 10 (47.6) | 7 (33.3) | 4 (19) | 4 (19) |
| Bravis, location Roosendaal | 98 | 42 (42.9) | 17 (17.3) | 11 (11.2) | 8 ( 8.2) |
| Canisius | 34 | 11 (32.4) | 7 (20.6) | 3 ( 8.8) | 4 (11.8) |
| Catharina | 82 | 30 (36.6) | 17 (20.7) | 12 (14.6) | 7 ( 8.5) |
| Diakonessenhuis | 10 | 7 (70) | 2 (20) | 2 (20) | 0 ( 0) |
| Elkerliek | 76 | 26 (34.2) | 12 (15.8) | 7 ( 9.2) | 6 ( 7.9) |
| Gelderse vallei | 114 | 36 (31.6) | 20 (17.5) | 10 ( 8.8) | 11 ( 9.6) |
| Haven | 10 | 2 (20) | 2 (20) | 2 (20) | 0 ( 0) |
| Ikazia | 70 | 38 (54.3) | 21 (30) | 21 (30) | 0 ( 0) |
| Maasstad | 102 | 44 (43.1) | 17 (16.7) | 15 (14.7) | 3 ( 2.9) |
| Maastricht UMC | 113 | 36 (31.9) | 16 (14.2) | 9 ( 8) | 8 ( 7.1) |
| Maxima | 89 | 36 (40.4) | 19 (21.3) | 10 (11.2) | 11 (12.4) |
| Meander MC | 51 | 25 (49) | 7 (13.7) | 6 (11.8) | 1 ( 2) |
| OLVG, location east | 113 | 34 (30.1) | 19 (16.8) | 19 (16.8) | 0 ( 0) |
| OLVG, location west | 85 | 34 (40) | 10 (11.8) | 10 (11.8) | 0 ( 0) |
| Rijnstate | 153 | 83 (54.2) | 31 (20.3) | 26 (17) | 13 ( 8.5) |
| Rivierenland | 12 | 5 (41.7) | 4 (33.3) | 1 ( 8.3) | 3 (25) |
| Rode Kruis | 43 | 21 (48.8) | 10 (23.3) | 7 (16.3) | 3 ( 7) |
| Franciscus Gasthuis | 51 | 22 (43.1) | 11 (21.6) | 8 (15.7) | 4 ( 7.8) |
| Spaarne Gasthuis Haarlem | 63 | 30 (47.6) | 12 (19) | 6 ( 9.5) | 7 (11.1) |
| Spaarne Gasthuis Hoofddorp | 22 | 11 (50) | 4 (18.2) | 3 (13.6) | 1 ( 4.5) |
| St. Anna | 40 | 15 (37.5) | 6 (15) | 2 ( 5) | 4 (10) |
| St. Antonius | 25 | 15 (60) | 6 (24) | 4 (16) | 3 (12) |
| UMC Utrecht | 10 | 3 (30) | 1 (10) | 1 (10) | 0 ( 0) |
| Vlietland | 47 | 14 (29.8) | 13 (27.7) | 9 (19.1) | 6 (12.8) |
| Amsterdam UMC, location VUMC | 91 | 45 (49.5) | 26 (28.6) | 12 (13.2) | 14 (15.4) |
| Zaans MC | 50 | 15 (30) | 11 (22) | 9 (18) | 2 ( 4) |
| Zuydergeleen | 33 | 13 (39.4) | 6 (18.2) | 6 (18.2) | 0 ( 0) |
| **p-value** |  | 0.001 | 0.677 | 0.247 | 0.001 |

| **Table S13: Sensitivity analysis for the association between culture-positive sepsis and mortality (positive rectum cultures excluded)** | | | |
| --- | --- | --- | --- |
|  | Unadjusted analysis | | |
|  | p | RR | 95% CI |
| 28-day mortality | 0.006 | 1.43 | 1.12-1.97 |
| 90-day mortality | <0.001 | 1.41 | 1.15-1.71 |
|  | Adjusted analysis ∞ | | |
| 28-day mortality | 0.005 | 1.52 | 1.14-2.01 |
| 90-day mortality | <0.001 | 1.52 | 1.21-1.90 |
| ∞ Adjusted for age, group allocation, antibiotics at home, hospital location, source of infection and total amount of blood cultures drawn | | | |

| **Table S14: Sensitivity analysis for the association between administration of pre-hospital antibiotics in the ambulance and mortality in the subgroup of patients with culture-positive sepsis (positive urine cultures excluded)** | | | |
| --- | --- | --- | --- |
|  | p | RR | 95% CI |
|  | 28-day mortality | | |
| Unadjusted analysis | 0.415 | 1.18 | 0.79-1.73 |
| Adjusted analysis ∞ | 0.584 | 1.10 | 0.79-1.51 |
|  | 90-day mortality | | |
| Unadjusted analysis | 0.489 | 1.15 | 0.77-1.70 |
| Adjusted analysis ∞ | 0.737 | 1.06 | 0.75-1.47 |
| ∞ Adjusted for ceftriaxone resistance and antibiotics at home | | | |

| **Table S15: 28-day and 90-day mortality for patients with the 10 most common pathogens in pre-hospital blood culture** | | | |
| --- | --- | --- | --- |
|  | | 28-day mortality | 90-day mortality |
| pathogen | n | n (%) | n (%) |
| E. coli | 73 | 8 (11) | 9 (12) |
| S. pneumoniae | 28 | 4 (14) | 5 (18) |
| S. aureus | 22 | 3 (14) | 7 (32) |
| Klebsiella  (not otherwise specified) | 13 | 2 (15) | 2 (15) |
| K. pneumoniae | 11 | 6 (55) | 6 (55) |
| Streptococcus  (not otherwise specified) | 11 | 2 (18) | 2 (18) |
| E. faecalis | 7 | 2 (29) | 3 (43) |
| P. mirabilis | 5 | 1 (20) | 2 (40) |
| P. aeruginosa | 5 | 3 (60) | 3 (60) |
| E. cloacae | 4 | 1 (25) | 1 (25) |

| **Table S16: 28-day and 90-day mortality for patients with the 10 most common pathogens in the in-hospital blood culture** | | | |
| --- | --- | --- | --- |
|  | | 28-day mortality | 90-day mortality |
| pathogen | n | n (%) | n (%) |
| E. coli | 145 | 10 (7) | 17 (12) |
| S. pneumoniae | 43 | 2 (5) | 2 (5) |
| S. aureus | 40 | 6 (15) | 9 (22) |
| Streptococcus (not otherwise specified) | 18 | 3 (17) | 3 (17) |
| P. mirabilis | 17 | 3 (18) | 3 (18) |
| E. faecalis | 15 | 5 (33) | 7 (47) |
| K. pneumoniae | 13 | 3 (23) | 3 (23) |
| P. aeruginosa | 12 | 1 (8) | 2 (17) |
| Klebsiella (not otherwise specified) | 9 | 2 (22) | 2 (22) |
| C. difficile | 5 | 1 (20) | 1 (20) |

| **Table S17: 28-day and 90-day mortality for patients with the 10 most common pathogens in the pre-hospital and/or in-hospital blood culture** | | | |
| --- | --- | --- | --- |
|  | | 28-day mortality | 90-day mortality |
| pathogen | n | n (%) | n (%) |
| E. coli | 213 | 17 (8) | 25 (12) |
| S. pneumoniae | 65 | 5 (8) | 6 (9) |
| S. aureus | 49 | 7 (14) | 13 (27) |
| Streptococcus (not otherwise specified) | 27 | 4 (15) | 4 (15) |
| K. pneumoniae | 24 | 9 (38) | 9 (38) |
| Klebsiella (not otherwise specified) | 21 | 3 (14) | 3 (14) |
| P. mirabilis | 20 | 3 (15) | 4 (20) |
| E. faecalis | 17 | 5 (29) | 8 (47) |
| P. aeruginosa | 16 | 3 (19) | 4 (25) |
| S. dysgalactiae | 7 | 0 (0) | 0 (0) |

| **Table S18: 28-day and 90-day mortality for patients with the 10 most common pathogens in the urine culture** | | | |
| --- | --- | --- | --- |
|  | | 28-day mortality | 90-day mortality |
| pathogen | n | n (%) | n (%) |
| E. coli | 251 | 14 (6) | 25 (10) |
| E. faecalis | 83 | 7 (8) | 16 (19) |
| P. aeruginosa | 51 | 1 (2) | 8 (16) |
| Klebsiella (not otherwise specified) | 38 | 3 (8) | 7 (18) |
| P. mirabilis | 31 | 3 (10) | 6 (19) |
| Enterococcus (not otherwise specified) | 17 | 2 (12) | 4 (24) |
| C. albicans | 16 | 3 (19) | 3 (19) |
| K. pneumoniae | 16 | 3 (19) | 3 (19) |
| S. aureus | 14 | 3 (21) | 5 (36) |
| E. faecium | 12 | 1 (8) | 3 (25) |

| **Table S19: 28-day and 90-day mortality for patients with the 10 most common pathogens in the sputum culture** | | | |
| --- | --- | --- | --- |
|  | | 28-day mortality | 90-day mortality |
| pathogen | n | n (%) | n (%) |
| S. pneumoniae | 27 | 0 (0) | 1 (4) |
| S. aureus | 26 | 5 (19) | 7 (27) |
| E. coli | 19 | 4 (21) | 7 (37) |
| P. aeruginosa | 19 | 3 (16) | 3 (16) |
| Aspergillus | 9 | 2 (22) | 2 (22) |
| Moraxella catarrhalis | 7 | 0 (0) | 0 (0) |
| Klebsiella (not otherwise specified) | 6 | 1 (17) | 2 (33) |
| S. maltophilia | 6 | 0 (0) | 1 (17) |
| Staphylococcus (not otherwise specified) | 5 | 2 (40) | 2 (40) |
| C. tropicalis | 4 | 0 (0) | 0 (0) |

| **Table S20: 28-day and 90-day mortality for patients with the 10 most common pathogens in the wound/ skin/ soft tissue culture** | | | |
| --- | --- | --- | --- |
|  | | 28-day mortality | 90-day mortality |
| pathogen | n | n (%) | n (%) |
| S. aureus | 34 | 4 (12) | 7 (21) |
| E. coli | 20 | 1 (5) | 4 (20) |
| Streptococcus (not otherwise specified) | 17 | 2 (12) | 3 (18) |
| P. aeruginosa | 14 | 1 (7) | 2 (14) |
| E. faecalis | 14 | 1 (7) | 2 (14) |
| C. albicans | 10 | 2 (20) | 4 (40) |
| P. mirabilis | 9 | 0 (0) | 0 (0) |
| Staphylococcus (not otherwise specified) | 8 | 0 (0) | 0 (0) |
| Enterococcus (not otherwise specified) | 6 | 2 (33) | 2 (33) |
| Klebsiella (not otherwise specified) | 6 | 1 (17) | 1 (17) |

| **Table S21: The association between a positive (blood) culture and mortality in the subgroup of patients fulfilling sepsis-3 criteria (qSOFA in the ambulance and/or ED ≥ 2)** | | | | |
| --- | --- | --- | --- | --- |
|  | p | RR | | 95% CI |
|  | Bacteremia (positive pre-hospital and/or in-hospital blood culture) | | | |
|  | Unadjusted analysis |  |  | |
| 28-day mortality | 0.258 | 1.27 | 0.83-1.85 | |
| 90-day mortality | 0.043 | 1.54 | 1.01-1.83 | |
|  | Adjusted analysis ∞ |  |  | |
| 28-day mortality | 0.137 | 1.44 | 0.88-2.21 | |
| 90-day mortality | 0.033 | 1.51 | 1.03-2.08 | |
|  | Culture-positive sepsis (with or without bacteremia) | | | |
|  | Unadjusted analysis |  |  | |
| 28-day mortality | 0.249 | 1.24 | 0.85-1.78 | |
| 90-day mortality | 0.136 | 1.25 | 0.93-1.65 | |
|  | Adjusted analysis ∞ |  |  | |
| 28-day mortality | 0.340 | 1.24 | 0.79-1.88 | |
| 90-day mortality | 0.231 | 1.25 | 0.86-1.74 | |
| ∞ Adjusted for age, group allocation, antibiotics at home, hospital location, source of infection and total amount of blood cultures drawn | | | | |

| **Table S22: Organ dysfunction stratified by qSOFA score** | | | | |
| --- | --- | --- | --- | --- |
|  |  | qSOFA* in ambulance and ED* <2 | qSOFA* in ambulance and/or ED* ≥2 | p |
| n |  | 1050 | 632 |  |
| Number of organs with dysfunction (%) | 0 | 470 (44.8) | 85 (13.4) | <0.001 |
|  | 1 | 387 (36.9) | 200 (31.6) |  |
|  | 2 | 135 (12.9) | 200 (31.6) |  |
|  | 3 | 47 ( 4.5) | 82 (13) |  |
|  | 4 | 7 ( 0.7) | 46 ( 7.3) |  |
|  | 5 | 4 ( 0.4) | 14 ( 2.2) |  |
|  | 6 | 0 ( 0) | 5 ( 0.8) |  |
| Used abbreviations: qSOFA = quick Sequential Organ Failure Assessment score, ED = emergency department | | | | |

| **Table S23: Overview of positive in-hospital blood cultures (contamination excluded)** | |
| --- | --- |
| In-hospital blood culture result | Number of patients with culture result |
| E. coli | 144 |
| S. pneumoniae | 42 |
| S. aureus | 40 |
| Streptococcus, not otherwise specified | 18 |
| P. mirabilis | 17 |
| E. faecalis | 15 |
| K. pneumoniae | 13 |
| P. aeruginosa | 12 |
| Klebsiella, not otherwise specified | 9 |
| E. faecium | 5 |
| B. fragilis | 4 |
| Enterococcus, not otherwise specified | 4 |
| Hemolytic streptococcus group G | 4 |
| S. dysgalactiae | 4 |
| C. albicans | 3 |
| S. agalactiae | 3 |
| S. oralis | 3 |
| Acinetobacter, not otherwise specified | 2 |
| Bacteroides thetaiotaomicron | 2 |
| C. koseri | 2 |
| E. cloacae | 2 |
| E. lenta | 2 |
| F. nucleatum | 2 |
| Hemolytic streptococcus group B | 2 |
| Hemolytic streptococcus group C | 2 |
| K. oxytoca | 2 |
| S. bovis | 2 |
| S. pyogenes | 2 |
| S. salivarius | 2 |
| Actinomyces, not otherwise specified | 1 |
| Aerococcus, not otherwise specified | 1 |
| A. faeciporci | 1 |
| A. urinae | 1 |
| B. diminuta | 1 |
| B. ovatus | 1 |
| C. jejuni | 1 |
| Cytomegaly virus | 1 |
| E. aerogenes | 1 |
| E. avium | 1 |
| Epstein Barr virus | 1 |
| E. catenaformis | 1 |
| Hepatitis A virus | 1 |
| Hemolytic streptococcus, not otherwise specified | 1 |
| Moraxella catarrhalis | 1 |
| Morganella morganii | 1 |
| Mycobacterium, not other wise specified | 1 |
| P. aenibacillus | 1 |
| Peptostreptococcus, not otherwise specified | 1 |
| P. multocida | 1 |
| P. vulgaris | 1 |
| R. mucilaginosa | 1 |
| R. ornithinolytica | 1 |
| S. anginosus | 1 |
| S. gordonii | 1 |
| S. intermedius | 1 |
| S. marcescens | 1 |
| S. mitis | 1 |
| S. parasanguins | 1 |
| S. pasteurii | 1 |

| **Table S24: Overview of positive pre-hospital blood cultures (contamination excluded)** | |
| --- | --- |
| Pre-hospital blood culture result | Number of patients with culture result |
| E. coli | 73 |
| S. pneumoniae | 28 |
| S. aureus | 21 |
| Klebsiella, not otherwise specified | 13 |
| K. pneumoniae | 11 |
| Streptococcus, not otherwise specified | 10 |
| E. faecalis | 7 |
| P. mirabilis | 5 |
| P. aeruginosa | 5 |
| E. cloacae | 4 |
| S. oralis | 4 |
| Streptococcus group A | 3 |
| S. dysgalactiae | 3 |
| S. pyogenes | 3 |
| V. parvula | 3 |
| A. urinae | 2 |
| Hemolytic streptococcus, not otherwise specifed | 2 |
| Hemolytic strepococcus group B | 2 |
| P. micra | 2 |
| S. bovis | 2 |
| S. intermedius | 2 |
| S. salivarius | 2 |
| A. baumannii complex | 1 |
| Acinetobacter, not otherwise specified | 1 |
| Actinomyces, not otherwise specified | 1 |
| A. schaallii | 1 |
| B. casei | 1 |
| B. fragilis | 1 |
| D. hominis | 1 |
| E. aerogenes | 1 |
| E. avium | 1 |
| E. lenta | 1 |
| Hemolytic streptococcus group C | 1 |
| Moraxella catarrhalis | 1 |
| N. meningitidis | 1 |
| P. aenibacillus | 1 |
| Peptococcus | 1 |
| P. hominis | 1 |
| Proteus, not otherwise specified | 1 |
| S. agalactiae | 1 |
| Salmonella | 1 |
| S. milleri | 1 |
| s. parasanguins | 1 |
| S. sanguinis | 1 |

| **Table S25: Overview of positive urine cultures** | |
| --- | --- |
| Urine culture result | Number of patients with culture result |
| E. coli | 251 |
| E. faecalis | 83 |
| P. aeruginosa | 50 |
| Klebsiella, not otherwise specified | 38 |
| P. mirabilis | 31 |
| Enterococcus, not otherwise specified | 17 |
| C. albicans | 16 |
| K. pneumoniae | 16 |
| S. aureus | 14 |
| E. faecium | 12 |
| Citrobacter, not otherwise specified | 11 |
| S. pneumoniae | 11 |
| staphylococccus, not otherwise specified | 11 |
| A. urinae | 9 |
| E. cloacae | 7 |
| S. epidermidis | 7 |
| C. glabrata | 6 |
| Enterobacter, not otherwise specified | 6 |
| Morganella morgannii | 6 |
| Streptococcus, not otherwise specified | 5 |
| coliform rod-shaped bacterium | 4 |
| Proteus, not otherwise specified | 4 |
| C. koseri | 3 |
| S. haemolyticus | 3 |
| K. oxytoca | 2 |
| R. ornithinolytica | 2 |
| S. maltophilia | 2 |
| S. marcescens | 2 |
| Legionella fast-test | 2 |
| Acinetobacter, not otherwise specified | 1 |
| A. sanguinicola | 1 |
| A. schaallii | 1 |
| Bifidobacterium, not otherwise specified | 1 |
| C. dubliniensis | 1 |
| C. jeikeium | 1 |
| C. parapsilosis | 1 |
| C. sakazakii | 1 |
| C. striatum | 1 |
| C. tropicalis | 1 |
| E. aerogenes | 1 |
| Gram-postive bacterium | 1 |
| Hemoloytic streptoccus, not otherwise specified | 1 |
| Leptospira | 1 |
| S. dysgalactiae | 1 |
| S. liquefaciens | 1 |
| S. pneumoniae fast-test | 1 |
| S. pyogenes | 1 |

| **Table S26: Overview of positive sputum cultures (contamination excluded)** | |
| --- | --- |
| Sputum culture result | Number of patients with culture result |
| S. pneumoniae | 27 |
| S. aureus | 26 |
| E. coli | 19 |
| P. aeruginosa | 19 |
| Aspergillus | 9 |
| Moraxella catarrhalis | 7 |
| Klebsiella, not otherwise specified | 6 |
| S. maltophilia | 6 |
| Staphylococcus, not otherwise specified | 5 |
| C. tropicalis | 4 |
| E. cloacae | 3 |
| Enterobacter, not otherwise specified | 3 |
| P. mirabilis | 3 |
| achromobacter, not otherwise specified | 2 |
| Acinetobacter, not otherwise specified | 2 |
| C. glabrata | 2 |
| S. dysgalactiae | 2 |
| S. marcescens | 2 |
| Streptococcus, not otherwise specified | 2 |
| C. dubliniensis | 1 |
| C. freundii | 1 |
| Chryseobacterium | 1 |
| Citrobacter, not otherwise specified | 1 |
| C. koseri | 1 |
| coliform rod-shaped bacterium | 1 |
| E. faecalis | 1 |
| E. faecium | 1 |
| Enterococcus, not otherwise specified | 1 |
| Gram-negative bacterium | 1 |
| Gram-positive bacterium | 1 |
| K. pneumoniae | 1 |
| Morganella morgannii | 1 |
| M. pneumoniae | 1 |
| Mycobacterium, not otherwise specified | 1 |
| P. alcaligenes | 1 |
| P. jiroveci | 1 |
| Raoultella, not otherwise specified | 1 |

| **Table S27: Overview of positive wound/ skin/ soft tissue cultures (contamination excluded)** | |
| --- | --- |
| Wound/ skin/ soft tissue culture result | Number of patients with culture result |
| S. aureus | 34 |
| E. coli | 21 |
| Streptococcus, not otherwise specified | 18 |
| P. aeruginosa | 16 |
| E. faecalis | 15 |
| Candida albicans | 10 |
| P. mirabilis | 9 |
| Staphylococcus, not otherwise specified | 8 |
| Enterococcus, not otherwise specified | 7 |
| Klebsiella, not otherwise specified | 6 |
| S. pyogenes | 5 |
| Hemolytic streptococcus, not otherwise specified | 4 |
| S. epidermidis | 4 |
| Actinomyces, not otherwise specified | 3 |
| B. fragilis | 3 |
| S. dysgalactiae | 3 |
| Herpes simplex virus type 1 | 3 |
| Bacteriodes thetaiotaomicron | 2 |
| C. striatum | 2 |
| E. cloacae | 2 |
| E. faecium | 2 |
| Peptostreptococcus, not otherwise specified | 2 |
| S. oralis | 2 |
| Hemolytic streptococcus group C | 2 |
| B. vulgatus | 1 |
| C. freundii | 1 |
| E. aerogenes | 1 |
| Enterobacter, not otherwise specified | 1 |
| K. pneumoniae | 1 |
| Propionibacterium, not otherwise specified | 1 |
| S. intermedius | 1 |
| S. pneumoniae | 1 |
| Citrobacter, not otherwise specified | 1 |
| C. krusei | 1 |
| E. catenaformis | 1 |
| Hemolytic streptococcus group B | 1 |
| P. micra | 1 |
| Respiratory syncytial virus | 1 |
| S. haemolyticus | 1 |

| **Table S28: Overview of positive ceroberospinal fluid cultures (contamination excluded)** | |
| --- | --- |
| Cerobrospinal fluid culture result | Number of patients with culture result |
| E. faecalis | 1 |
| S. gordonii | 1 |
| S. milleri | 1 |
| S. pneumoniae | 1 |
| S. sanguinis | 1 |
| Streptococcus, not otherwise specified | 1 |

| **Table S29: Overview of positive pleural fluid cultures** | |
| --- | --- |
| Pleural fluid culture result | Number of patients with result |
| S. pneumoniae | 3 |
| C. albicans | 1 |
| S. intermedius | 1 |
| Streptococcus, not otherwise specified | 1 |

| **Table S30: Overview of positive broncho-alveolar lavage (BAL) cultures** | |
| --- | --- |
| BAL culture result | Number of patients with result |
| Aspgergillus | 3 |
| C. albicans | 3 |
| E. coli | 1 |
| Klebsiella, not otherwise specified | 1 |
| P. jiroveci | 1 |
| P. aeruginosa | 1 |
| S. pneumoniae | 1 |

| **Table S31: Overview of positive ascites cultures** | |
| --- | --- |
| Ascites culture result | Number of patients with result |
| E. coli | 2 |
| Citrobacter, not otherwise specified | 1 |
| E. cloacae | 1 |
| E. faecalis | 1 |
| E. faecium | 1 |
| Enterobacter, not otherwise specified | 1 |
| P. aeruginosa | 1 |
| S. haemolyticus | 1 |
| S. pneumoniae | 1 |

| **Table S32: Overview of joint puncture cultures** | |
| --- | --- |
| Joint puncture culture result | Number of patients with result |
| streptococcus, not otherwise specified | 2 |
| M. luteus | 1 |
| S. agalactiae | 1 |
| S. aureus | 1 |
| S. dysgalactiae | 1 |
| S. pneumoniae | 1 |

| **Table S33: Overview of positive intra-corporal material cultures** | |
| --- | --- |
| Intra-corporal material culture result | Number of patients with result |
| E. coli | 3 |
| E. faecalis | 2 |
| S. epidermidis | 2 |
| B. vulgatus | 1 |
| Enterococcus, not otherwise specified | 1 |
| Klebsiella, not otherwise specified | 1 |
| P. micra | 1 |
| Staphylococcus, not otherwise specified | 1 |

| **Table S34: Overview of positive feces cultures** | |
| --- | --- |
| Feces culture result | Number of patients with result |
| C. difficile | 12 |
| Noro virus | 8 |
| C. jejuni | 7 |
| E. coli | 4 |
| Salmonella | 4 |
| P. aeruginosa | 2 |
| Y. enterocolitica | 1 |

| **Table S35: Overview of positive pharynx cultures (including influenza swab)** | |
| --- | --- |
| Influenza A | 30 |
| C. albicans | 22 |
| Influenza B | 17 |
| Rhino virus | 7 |
| S. aureus | 6 |
| E. coli | 5 |
| P. aeruginosa | 5 |
| Gram-positive bacterium, not otherwise specified | 3 |
| C. glabrata | 3 |
| Respiratory syncytial virus | 2 |
| C. psittaci | 2 |
| E. faecium | 2 |
| Human metapneumo virus | 2 |
| M. pneumoniae | 2 |
| Paenibacillus, not otherwise specified | 1 |
| S. hominis | 1 |
| S. lugdunensis | 1 |
| Aspergillus | 1 |
| C. sputigena | 1 |
| Epstein Barr virus | 1 |
| E. faecalis | 1 |
| Enterobacter, not otherwise specified | 1 |
| Herpes simplex virus type 1 | 1 |
| Morganella morgannii | 1 |
| S. marcescens | 1 |
| S. pyogenes | 1 |
| streptococcus, not otherwise specified | 1 |
| virus, not otherwise specified | 1 |

| **Table S36: Overview of positive rectum cultures** | |
| --- | --- |
| Rectum culture result | Number of patients with result |
| E. coli | 16 |
| C. albicans | 8 |
| Enterobacter, not otherwise specified | 3 |
| Klebsiella, not otherwise specified | 3 |
| P. aeruginosa | 3 |
| C. glabrata | 2 |
| E. faecalis | 2 |
| Enterococcus, not otherwise specified | 2 |
| Citrobacter, not otherwise specified | 1 |
| E. faecium | 1 |
| S. marcescens | 1 |
| Staphylococcus, not otherwise specified | 1 |

| **Table S37: Overview of positive perineum cultures** | |
| --- | --- |
| Perineum culture result | Number of patients with result |
| E. coli | 2 |
| C. albicans | 1 |
| C. tropicalis | 1 |
| P. aeruginosa | 1 |
| S. aureus | 1 |

**References**

1. Hall KK, Lyman JA. Updated review of blood culture contamination. Clin Microbiol Rev. 2006;19(4):788-802.

2. Baum GL. The significance of Candida albicans in human sputum. N Engl J Med. 1960;263:70-3.

3. Robinson J. Colonization and infection of the respiratory tract: What do we know? Paediatr Child Health. 2004;9(1):21-4.

4. Levy MM, Fink MP, Marshall JC, Abraham E, Angus D, Cook D, et al. 2001 SCCM/ESICM/ACCP/ATS/SIS International Sepsis Definitions Conference. Intensive Care Med. 2003;29(4):530-8.
